# Immunological parameters as biomarkers of response to MicroCrystalline Tyrosine-adjuvanted mite immunotherapy^☆^

**DOI:** 10.1016/j.waojou.2021.100545

**Published:** 2021-06-06

**Authors:** José L. Justicia, Clara Padró, Albert Roger, Francisco Moreno, Manuel J. Rial, Antonio Parra, Antonio Valero, Alfons Malet, Aina Teniente, Anna Boronat, Carla Torán-Barona

**Affiliations:** a Allergy Therapeutics Ibérica, Sant Joan Despí, Barcelona, Spain; b Allergy Department, Germans Trias i Pujol Hospital, Badalona, Spain; c Dr. Lobatón Clinic. Cádiz, Spain; d Allergy Department, Complexo Hospitalario Universitario A Coruña, A Coruña, Spain; e Pneumonology and Respiratory Allergy, Hospital Clinic de Barcelona, IDIBAPS, CIBERES, Barcelona, Spain; f Al·lergo Centre, Barcelona, Spain; g Immunology Division, Germans Trias i Pujol Hospital, Badalona, Spain; h Department of Cellular Biology, Physiology and Immunology, Universitat Autònoma de Barcelona, Barcelona, Spain

**Keywords:** House dust mite, Allergic rhinitis, Subcutaneous allergen immunotherapy, Microcrystalline Tyrosine, Immunological biomarkers, Sensitization profile, Allergoid, Adjuvant

## Abstract

**Background:**

Despite the effectiveness of allergen immunotherapy (AIT), some patients are unresponsive for reasons still unknown; yet validated response biomarkers remain unavailable.

**Objective:**

To analyze immunological parameters as biomarkers to monitor and predict clinical response to a MicroCrystalline Tyrosine-adjuvanted house dust mite (HDM) AIT in patients with allergic rhinitis (AR).

**Methods:**

Observational, prospective, multicenter study including adult patients (aged 18–65 years) with AR, with and without asthma, sensitized to the HDM *Dermatophagoides pteronyssinus* (DP) and prescribed Acarovac Plus® DP 100% in the routine practice. Serum concentrations of total IgE, specific IgE, specific IgG4, IL-4, IL-5, IL-10, IL-13, and IFN-γ were compared between baseline and 12 months after AIT. The relationship between patients’ baseline immunological profiles and classification as low, high, and non-responders and between their sensitization profile to DP allergens and effectiveness were analyzed.

**Results:**

Of 141 patients recruited, 118 (mean [SD] age of 33.6 [9.5] years) were evaluable. One year after treatment, Der p 1-specific IgE, DP-specific IgG4, and IL-10 increased by a mean (SD) of 3.4 (13.6) kU/L (*p* = 0.016), 0.43 (0.55) mg/L (*p* < 0.0001), and 1.35 (7.56) pg/mL (*p* = 0.033), respectively. Non-responders showed increased baseline levels of IL-13 compared to high responders (*p* = 0.037). Changes in effectiveness variables between baseline and after AIT were similar regardless of the sensitization profile.

**Conclusion:**

Non-responsive patients to AIT showed increased baseline IL-13 concentrations, suggesting its value as prognostic biomarker. DP-specific AIT increased Der p 1-specific IgE, DP-specific IgG4, and IL-10 concentrations in patients with AR. All patients benefited from treatment regardless of their sensitization profile to major DP allergens.

## Introduction

Allergic rhinitis (AR) is one of the most prevalent allergic diseases, with an estimated prevalence of 10%–40%.^1^ Its high symptom burden, together with that of its associated allergic conditions, such as asthma, impact patients’ quality of life and daily activities, and generating high direct and indirect costs.^1^^,^^2^ Of the multiple allergens involved in AR, mites are one of the most frequent, and, in Spain, they are the second cause of AR after pollen.^3^^,^^4^ The house dust mite (HDM) *Dermatophagoides pteronyssinus* (DP) is a common indoor allergen comprising at least 23 different variants, of which Der p 1 and Der p 2 are the most sensitizing.^5^, ^6^, ^7^

Effective management of AR entails adequate symptom control and, to this end, current guidelines recommend allergen avoidance and pharmacological treatment.^2^^,^^8^ However, in many patients conventional pharmacological treatment may be insufficient; it induces side effects and fails to provide long-term benefits.^9^ In contrast, allergen immunotherapy (AIT) targets the underlying cause of the disease by inducing immune tolerance to specific allergens, providing long-term effects and potentially modifying the course of the disease.^10^, ^11^, ^12^ Despite the growing evidence about the clinical benefits of AIT, the degree of clinical response varies, being suboptimal in some patients for reasons still unknown.^13^^,^^14^ In this context, understanding the immunological mechanisms of immune tolerance induced by AIT may enable to identify biomarkers associated with clinical response.

Despite the growing knowledge about the immunological mechanisms of AIT, investigators claim the availability of validated biomarkers to monitor and predict the efficacy of AIT treatments at an individual level with the goal of improving patients’ clinical management.^15^, ^16^, ^17^ In this sub-analysis of the immunological results obtained in a previous prospective study,^18^ we assessed the changes observed in immunological parameters in response to subcutaneous AIT (SCIT) with Acarovac Plus® and evaluated the baseline immunological profile to identify predictive biomarkers of clinical response. In addition, we analyzed the effectiveness of Acarovac Plus® in patients with different predominant profiles of sensitization to major DP allergens.

## Material and methods

### Study design and population

This observational, prospective, multicenter study was a sub-analysis of the immunological variables obtained from a study in adult patients (aged 18–65 years) diagnosed with allergic rhinitis for ≥ 1 year, with and without allergic asthma, caused by sensitization to HDM DP.^18^ A total of 141 patients, who attended visits at 10 Spanish allergy centers between June 2015 and June 2016 and who were prescribed AIT with Acarovac Plus® DP 100% according to the routine practice, were consecutively included in the study.

Acarovac Plus® is a purified allergen extract from the HDM DP modified with glutaraldehyde and associated with MicroCrystalline Tyrosine (MCT) as an adjuvant. Treatment with Acarovac Plus® was carried out in 2 phases: an up-dosing phase consisting of 4 injections of 0.05, 0.1, 0.3, and 0.5 mL at one-to two-week intervals and a maintenance phase of 8 injections of 0.5 mL at six-week intervals. Treatment administration started at Visit 1 (V1), and assessments were performed during the Selection Visit (V0), V1 (4 weeks after V0), Visit 2 (V2) (6 ± 1 months after V1), and Final Visit (FV) (12 ± 1 months after V1). The total duration of the study was 13 months. All participating patients provided written informed consent during the Selection Visit (V0). The study was conducted in accordance with the Helsinki Declaration and the local Personal Data Protection Law (LOPD 15/1999); the study protocol was approved by the ethics committee of Hospital Germans Trias i Pujol (Barcelona, Spain) (EPA-14-023).

### Variables and clinical assessments

Demographic and clinical variables, including history of allergic disease, previous and concomitant diseases, and concomitant medications were recorded from patients’ medical records during V0. Patients were also provided with a diary to record their symptoms (presence and intensity) and use of medication to treat allergy symptoms for 4 weeks prior to V1, V2, and FV, and investigators collected the information at the corresponding visit. Additionally, patients with asthma underwent a functional respiratory test (spirometry) during V0. The primary endpoint of this study was to evaluate the effectiveness of AIT by assessing changes between V1 (ie, symptoms and use of medication during the 4 weeks between V0 and V1) and FV (ie, symptoms and use of medication during the 4 weeks prior to FV) in the Combined Symptom and Medication Score (CSMS), a 0-6-point scale described by the European Academy of Allergy and Clinical Immunology.^19^ Additional effectiveness variables assessed, included daily symptom and medication scores, “well” and “bad” days, clinical evaluation of symptoms, and Nasal Provocation Test results.^18^

### Immunological assessments

Serum concentrations of total IgE, specific IgE, specific IgG4, and cytokines, including IL-4, IL-5, IL-10, IL-13 and IFN-γ, were determined from blood samples obtained at V0 and FV. DP-specific IgE (DP-sIgE), Der p 1-specific IgE (Der p 1-sIgE), Der p 2-specific IgE (Der p 2-sIgE), and DP-specific IgG4 (DP-sIgG4) were determined according to the routine clinical practice using ImmunoCAP® (Thermo Fisher Scientific, Massachusetts, USA), whereas cytokines were determined by cytometric bead array (BD Biosciences).

### Statistical analysis

In order to analyze treatment effectiveness according to patients’ predominant sensitization (Der p 1 vs. Der p 2), patients were categorized based on 7 classes of serum concentrations of specific IgE to Der p 1 or Der p 2 (ie, Class 0: <0.35; Class 1: 0.35–0.70; Class 2: 0.71–3.50; Class 3: 3.51–17.50; Class 4: 17.51–50.0; Class 5: 50.1–100; Class 6: >100 kU/L), allowing the definition of 3 groups: (1) Predominant Sensitization to Der p 1 (PS to Der p1), defined by Der p 1-sIgE class > Der p 2-sIgE class; (2) Predominant Sensitization to Der p 2 (PS to Der p 2), defined by Der p 2-sIgE class > Der p 1-sIgE class; and (3) Same Class/No Predominant Sensitization (SC/NP), defined by equal sensitization to Der p 1 and Der p 2.

In order to screen the immunological profile to identify immunological parameters as predictive biomarkers of treatment response, patients with available data regarding baseline serum values of immunoglobulins and cytokines and changes in CSMS one year after AIT were categorized according to their response (ie, CSMS reduction). Patients with a CSMS reduction <20%, 20%–50%, and ≥50% were categorized as non-responders (NRs), low responders (LRs), and high responders (HRs), respectively.

Patients who met the selection criteria and received at least 1 treatment dose were evaluated. Categorical variables were described as frequencies and percentages, and quantitative variables as the mean and standard deviation (SD) and/or the median and interquartile range (IQR). Categorical variables were compared using the McNemar test and Fisher's test and quantitative variables were compared using the non-parametric Mann-Whitney U and Wilcoxon tests, for paired and unpaired data, respectively. The baseline immunological parameters among the different response groups were compared using the one-way ANOVA test and a multivariate analysis (logistic regression), including the variables that yielded significant *p* values in the bivariate analysis. ROC curves were used to assess the ability of baseline IL-13 levels to predict response to AIT treatment by measuring their area under the receiver-operating curve (AUC). The significance threshold for all bivariate and multivariate analyses was set at a two-sided α = 0.05. All analyses were performed using the statistical package support PASS 2011 version and Prism GraphPad v6.0.

## Results

### Demographic, clinical and treatment characteristics of study patients

Of 141 patients initially recruited, 12 failed to meet the inclusion/exclusion criteria and 11 remained untreated, resulting in an evaluable study population of 118 patients with a mean (SD) age of 33.6 (9.5) years.^18^ The demographic, clinical, and treatment characteristics of the study population are summarized in Table 1. Mean (SD) time of rhinitis evolution was 11.72 (10.15) and 14.11 (18.77) years for the complete study population and the 55 patients with rhinitis and asthma, respectively.

**Table 1:** Demographic, clinical, and treatment characteristics of study patients, *n (%*) N = 118

**Demographic Characteristics**	

Sex	
Male	51 (43.2)
Female	67 (56.8)
**Clinical Characteristics**	
Asthma diagnosis	55 (46.6)
Other allergies	3 (2.5)
Food allergy	1 (0.8)
Conjunctivitis	1 (0.8)
**Treatment Characteristics**	
Previous immunotherapy	2 (1.7)
Immunotherapy for mites	1 (0.8)
Medication for allergic rhinitis	103 (87.3)
Oral antihistamines	92 (89.3)
Nasal corticosteroids	71 (68.9)
Other concomitant medication	36 (30.5)

### Immunological changes in response to AIT

**Fig. 1:**
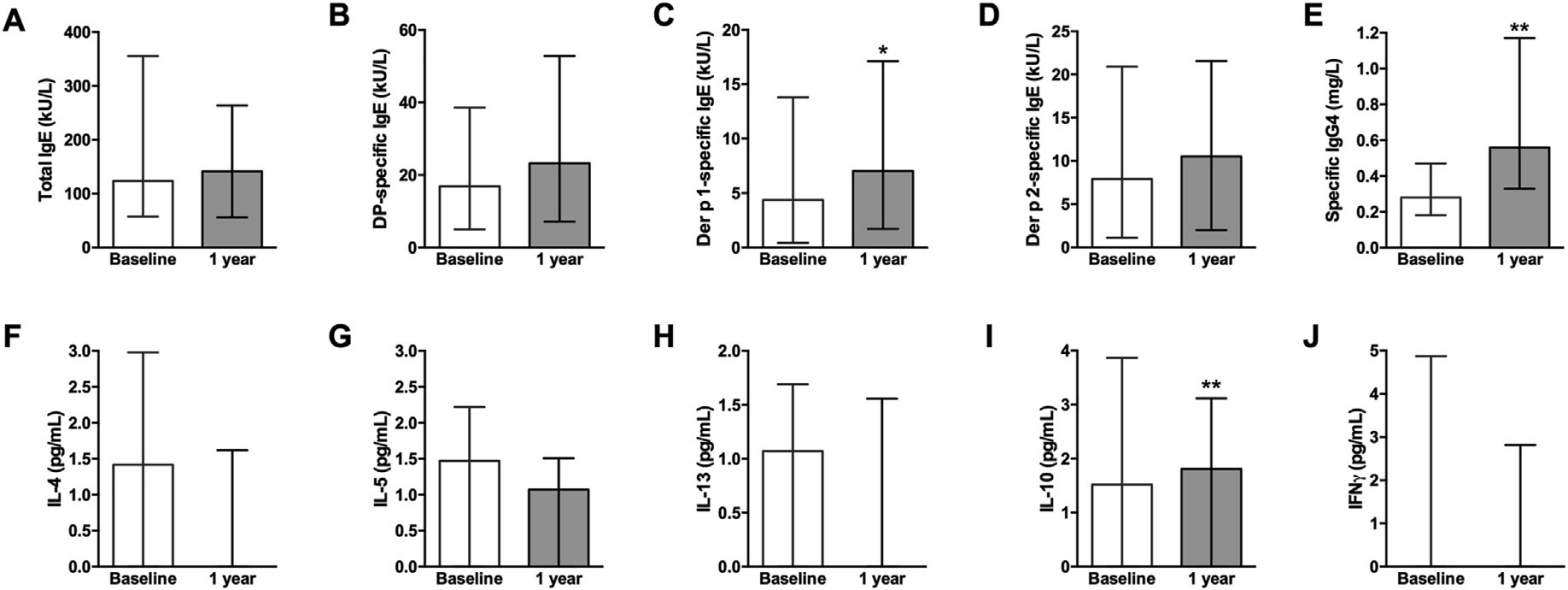
Serum concentrations of total IgE (A), DP-sIgE (B), Der p 1-sIgE (C), Der p 2-sIgE (D), DP-sIgG4 (E), IL-4 (F), IL-5 (G), IL-13 (H), IL-10 (I), and IFNγ (J) at the indicated timepoints. Columns and error bars represent the median and interquartile range, respectively. Wilcoxon test ∗*p* < 0.05, ∗∗*p* < 0.01

Of the immunoglobulins analyzed, Der p 1-sIgE and DP-sIgG4 significantly increased by a mean (SD) of 3.4 (13.57) kU/L (n = 67, *p* = 0.0160) and 0.43 (0.55) mg/L (n = 68, *p* < 0.0001), respectively, between baseline and 1 year after treatment (Fig. 1C, E). The concentration of DP-sIgE showed a trend towards an increase from a mean (SD) of 26.5 (27.5) to 32.8 (31.6) kU/L, albeit not statistically significant (n = 67, *p* = 0.092), whereas Der p 2-sIgE and total IgE (tIgE) remained unchanged 1 year after AIT (n = 67 for both, *p* = 0.297 and *p* = 0.859, respectively) (Fig. 1A, B, D). Regarding cytokines, IL-10 significantly increased by a mean (SD) of 1.35 (7.56) pg/mL (n = 65, *p* = 0.033) between baseline and 1 year after treatment and IL-5 showed a trend to decrease, albeit not significantly (*p* = 0.065); whereas concentrations of IL-4, IL-13, and IFN-γ remained unchanged (n = 65 for all, *p* = 0.216, *p* = 0.982, and *p* = 0.841, respectively) (Fig. 1F–J).

### Immunological profile as biomarker of treatment response

**Fig. 2:**
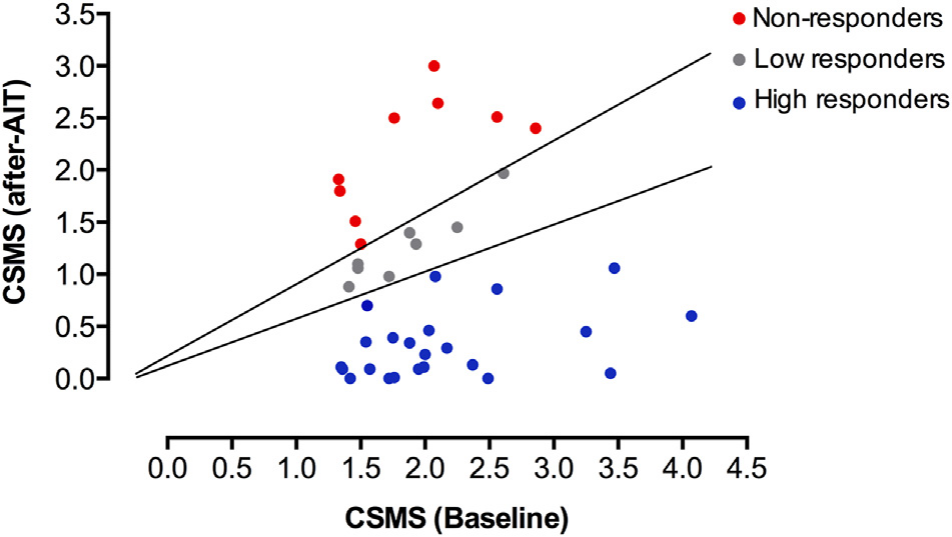
Categorization of patients according to their response. Each data point corresponds to a patient with severe disease (CSMS ≥1.33). Lines separate patients' categories according to treatment response, measured as the relative decrease of CSMS after AIT. AIT, Allergen immunotherapy; CSMS, Combined Symptom and Medication Scores

**Fig. 3:**
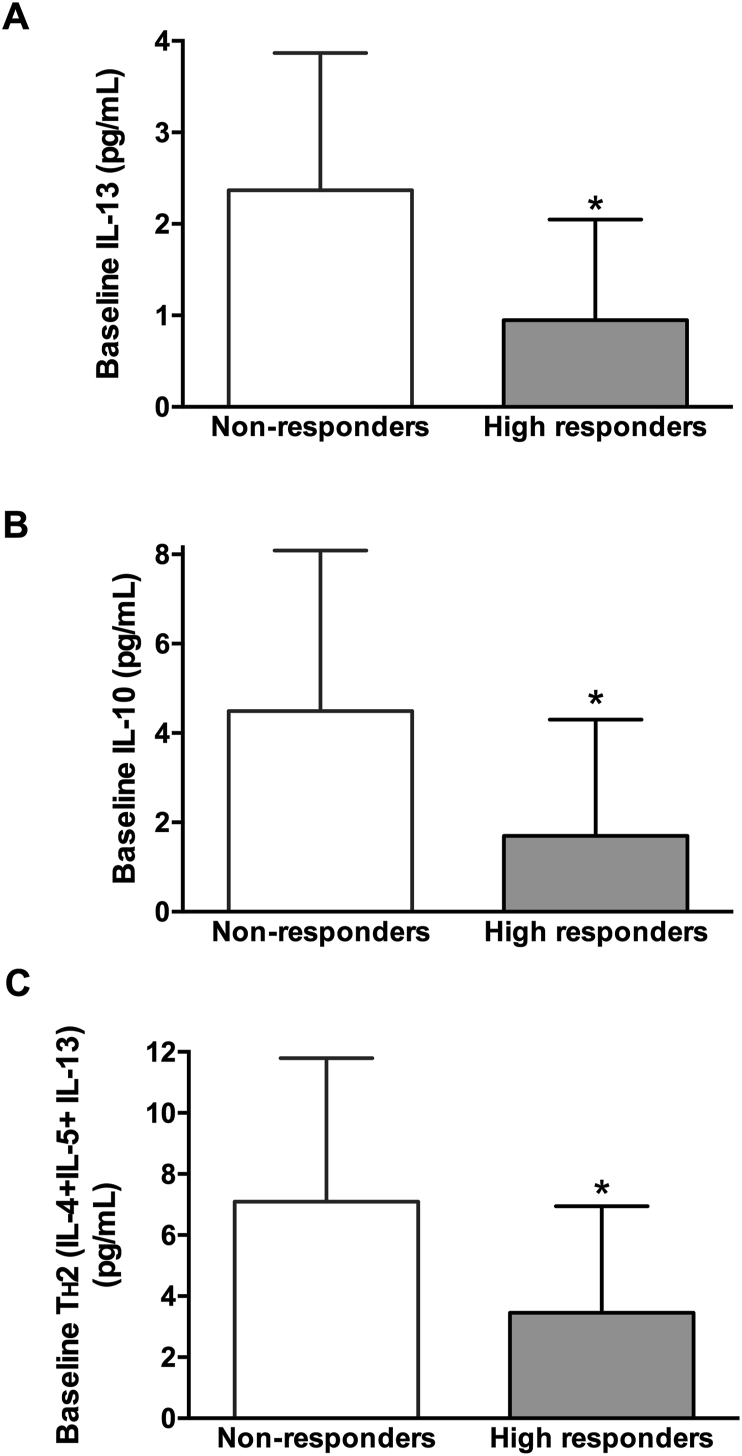
Baseline serum concentrations of IL-13 (A), IL-10 (B), and T_H_2 (IL-4 + IL-5 + IL-13) according to clinical response in patients with severe disease. Columns and error bars represent the mean and SD, respectively. Non-responders, n = 6 and high responders, n = 22. SD, standard deviation ∗One-way ANOVA *p* < 0.05 for comparisons between groups

Of the 118 patients included in the study, 79 had available data for changes in CSMS between baseline and 1 year after AIT as well as baseline serum levels of immunoglobulins (tIgE, DP-sIgE, DP-sIgG4, Der p 1-sIgE, and Der p 2-sIgE) and cytokines (IL-4, IL-5, IL-13, IL-10 and IFN-γ), and were categorized according to their response as NRs, LRs, and HRs. A comparison of the baseline immunological parameters of NRs and HRs showed no significant differences between them. Likewise, the subset of patients with more severe disease (n = 40), identified as those with a baseline CSMS ≥1.33—median baseline CSMS in the study population—were categorized as NRs (n = 8), LRs (n = 9), and HRs (n = 23) according to their clinical response (Fig. 2). Of those, 32 patients had available baseline data for all cytokines (NRs, n = 6; LRs, n = 4; HRs, n = 22) and were included in univariate and bivariate analyses. Baseline serum levels of IL-13, IL-10, and T_H_2 profile (combined IL-4, IL-5, and IL-13) were significantly higher in NRs compared to HRs (*p* = 0.019, *p* = 0.043, and *p* = 0.044, respectively, One-way ANOVA) (Fig. 3, Table 2). The remaining absolute and relative baseline immunological parameters evaluated, including tIgE, DP-sIgE, Der p 1-sIgE, Der p 2-sIgE, Der p 1+p 2-sIgE, DP-sIgG_4_, DP-sIgE/tIgE, Der p1-sIgE/tIgE, Der p2-sIgE/tIgE, Der p 1+p 2-sIgE/tIgE, Der p 1-sIgE/DP-sIgE, Der p 2-sIgE/DP-sIgE, Der p 1 + p 2-sIgE/DP-sIgE, DP-sIgG4/DP-sIgE, IL-4, IL-5, IFN-ɣ, T_H_1 (IFN-ɣ + IL-10), and T_H_1/T_H_2, showed no significant differences between response categories (Table 2). The 3 immunological parameters that yielded significant differences were included in a multivariate analysis, being baseline IL-13 levels the only parameter that was statistically significant (*p* = 0.037). Consequently, patients with higher baseline IL-13 levels were less likely to respond to AIT (OR = 0.475, 95% CI 0.236–0.957). The ROC analysis of baseline IL-13 levels showed and AUC of 0.788 (95% CI:0.562–1.01) (*p* = 0.033). Regarding the sensitivity and specificity of IL-13 as prognosis factor of response, the most sensitive and specific cut-off was 1.335, showing a specificity of 0.83 and a sensitivity of 0.73.

**Table 2:** Baseline immunological parameters according to treatment response, *mean (SD)*.

	Non-Responders n = 6	High Responders n = 22	*P* Value^a^
tIgE (kU/L)	326.2 (414.2)	164.9 (209.8)	0.193
DP-sIgE (kU/L)	25.08 (18.26)	22.22 (23.11)	0.782
DP-sIgG4 (mg/L)	0.46 (0.30)	0.31 (0.23)	0.179
Der p 1-sIgE (kU/L)	9.90 (8.85)	8.63 (12.07)	0.812
Der p 2-sIgE (kU/L)	9.61 (6.94)	11.73 (12.71)	0.700
Der p 1+ p 2-sIgE (kU/L)	19.52 (14.24)	20.36 (23.50)	0.935
DP-sIgE/tIgE	17.66 (16.98)	18.38 (11.77)	0.906
Der p 1-sIgE/tIgE	6.00 (7.56)	6.04 (5.13)	0.985
Der p 1-sIgE/DP-sIgE	31.64 (22.87)	31.19 (18.82)	0.961
Der p 2-sIgE/tIgE	8.08 (10.91)	10.24 (9.05)	0.622
Der p 2-sIgE/DP-sIgE	33.17 (21.69)	52.91 (38.31)	0.241
Der p 1+ p 2-sIgE/tIgE	14.07 (15.33)	16.29 (11.14)	0.693
Der p 1 + p 2-sIgE/DP-sIgE	64.81 (34.70)	84.11 (41.94)	0.312
DP-sIgG4/DP-sIgE	30.23 (23.87)	36.25 (41.81)	0.740
IL-4 (pg/ml)	2.92 (2.48)	1.43 (1.56)	0.080
IL-5 (pg/ml)	1.80 (1.42)	1.06 (1.09)	0.178
IL-13 (pg/ml)	2.37 (1.54)	0.95 (1.15)	**0.019**
IL-10 (pg/ml)	4.49 (3.62)	1.70 (2.62)	**0.043**
IFN-ɣ (pg/ml)	3.68 (4.55)	1.50 (3.23)	0.191
TH1 (IFN-ɣ + IL-10) (pg/ml)	8.17 (8.07)	3.20 (5.71)	0.095
TH2 (IL-4 + IL-5 + IL-13) (pg/ml)	7.10 (4.73)	3.45 (4.73)	**0.044**
TH1/TH2 Ratio	0.95 (0.40)	0.76 (0.75)	0.565

Abbreviations: DP, *Dermatophagoides pteronyssinus*; sIgE, specific IgE; sIgG4, specific IgG4; tIgE, total IgE.

a One-way ANOVA. Statistically significant differences are shown in bold type.

### Treatment effectiveness according to predominant sensitization

Of the 99 patients with available data at baseline for specific IgE to Der p 1 and Der p 2, 12 (12.1%) were not sensitized to Der p 1 or Der p 2 (specific IgE <0.35 kU/L, class 0), 5 (5.1%) patients were sensitized to Der p 1 but not to Der p 2, 12 (12.1%) patients were sensitized to Der p 2 but not to Der p 1, and 70 (70.7%) patients were sensitized to Der p 1 and Der p 2 (Table 3). Regarding the sensitization groups, 11 (11.1%) and 37 (37.4%) patients were predominantly sensitized to Der p 1 and Der p 2, respectively, whereas 51 (51.5%) patients showed no predominant sensitization (i.e., equal sensitization to Der p 1 and Der p 2) and were included in the Same Class/No Predominant Sensitization (SC/NP) group (Table 3). At baseline, CSMS, daily symptom score, % of well and bad days, NPT symptom score, and ESPRINT-15 questionnaire scores were similar between groups, whereas daily medication score, NPT (% drop of NIPF), and visual analog scale (VAS) scores showed statistically significant differences between groups (Table S1). The distribution of patients according to baseline rhinitis classification, including frequency (persistent or intermittent), severity (mild and moderate/severe), and control (not controlled, partially controlled, and controlled) was similar between predominant sensitization groups (Fig. S1).

**Table 3:** Distribution of patients according to baseline levels of specific IgE for Der p 1 and Der p 2, *n (%*) n = 99

Der p 2	Der p 1					
	Class 0	Class 1	Class 2	Class 3	Class 4	Class 5
Class 0	12 (12.12)	0 (0)	1 (1.01)	3 (3.03)	1 (1.01)	0 (0)
Class 1	2 (2.02)	0 (0)	1 (1.01)	0 (0)	0 (0)	0 (0)
Class 2	3 (3.03)	1 (1.01)	7 (7.07)	1 (1.01)	0 (0)	0 (0)
Class 3	5 (5.05)	0 (0)	10 (10.1)	19 (19.19)	3 (3.03)	0 (0)
Class 4	0 (0)	0 (0)	0 (0)	12 (12.12)	10 (10.1)	1 (1.01)
Class 5	2 (2.02)	0 (0)	0 (0)	0 (0)	2 (2.02)	3 (3.03)

Changes in the effectiveness variables 1 year after AIT were assessed in those patients with available data, were similar between the predominant sensitization groups and showed no significant differences, suggesting that treatment with the DP allergoid was effective regardless of the predominant sensitization (Table 4). Remarkably, the 12 patients who were not sensitized to Der p 1 and Der p 2 showed a mean (SD) reduction in CSMS of 0.60 (0.89), indicating a clinical benefit in patients sensitized to DP allergens other than Der p 1 and Der p 2. Similar to the quantitative variables, the distribution of patients according to rhinitis frequency, severity, and control one year after AIT was similar among the three groups (Fig. S1).

**Table 4:** Change in effectiveness variables according to predominant sensitization, *mean (SD)*.

	Change Baseline - One Year After AIT			*P* Value^a^		
	Der p 1 n = 11^b^	Der p 2 n = 37	SC/NP n = 51	Der p 1 vs. Der p 2	Der p 1 vs. SC/NP	Der p 2 vs. SC/NP
CSMS n = 66	−1.09 (1.22) *n=8*	−0.58 (1.37) *n=28*	−0.79 (1.05) *n=30*	0.332	0.400	0.815
Daily symptom score n = 67	−0.31 (0.79) *n=8*	−0.23 (0.81) *n=29*	−0.53 (0.69) *n=30*	0.897	0.485	0.187
Daily medication score n = 72	−0.69 (0.71) *n=9*	−0.37 (0.86) *n=29*	−0.24 (0.60) *n=34*	0.354	0.102	0.259
% Well days n = 73	26.6 (54.5) *n=9*	14.6 (44.5) *n=30*	15.1 (46.6) *n=34*	0.368	0.570	0.995
% Bad days n = 69	−4.76 (34.4) *n=9*	−0.74 (26.7) *n=29*	−6.68 (18.3) *n=31*	0.509	0.879	0.408
ESPRINT-15 global score n = 69	−1.63 (0.92) *n=8*	−1.04 (1.90) *n=28*	−1.58 (1.64) *n=33*	0.181	0.683	0.155
Visual Analog Scale score n = 71	−3.33 (1.65) *n=9*	−4.41 (3.29) *n=28*	−3.49 (2.74) *n=34*	0.367	0.709	0.258
NPT (% drop of nasal inspiratory peak flow) n = 52	−23.2 (23.7) *n=5*	−12.5 (42.2) *n=23*	−8.0 (28.0) *n=24*	0.764	0.285	0.516
NPT (symptom score) n = 53	−1.33 (2.42) *n=6*	−2.09 (2.78) *n=23*	−2.08 (2.47) *<n=24*	0.765	0.773	0.949

CSMS, combined symptom and medication score; NP, non-predominant sensitization; NPT, nasal provocation test; SC/NP, same class/no predominant sensitization; SD, standard deviation.

a Mann-Whitney.

b Total number of patients per group; patients with available data are indicated in the corresponding cell

**Fig. 4:**
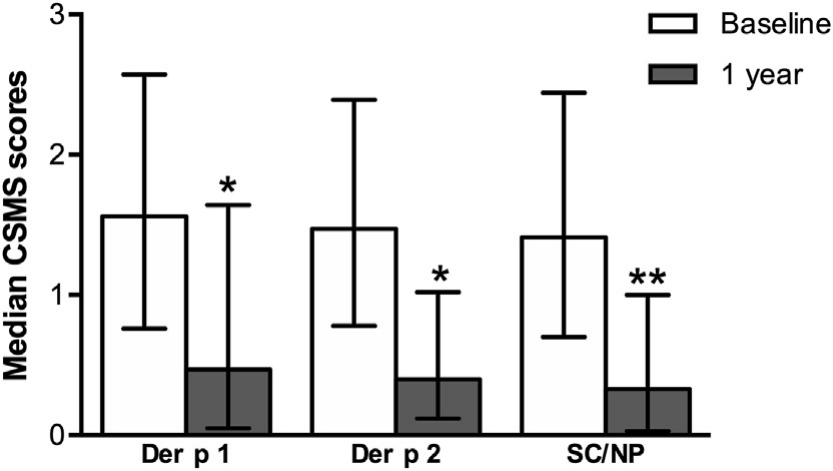
Combined symptom and medication score (CSMS) according to predominant sensitization at baseline. Columns and error bars represent the median and interquartile range, respectively. SC/NP, same class/no predominant sensitization. Wilcoxon test *∗p* < 0.05 and ∗∗*p* < 0.01, for differences between timepoints

Regarding the evolution of effectiveness variables within each predominant sensitization group, CSMS, the primary endpoint of this study, significantly decreased in the 3 predominant sensitization groups (Fig. 4).

## Discussion

This prospective analysis of immunological parameters in 118 patients sensitized to the HDM DP provided evidence regarding the immunological changes induced by AIT with Acarovac Plus®, showing increased levels of Der p 1-sIgE, DP-sIgG4, and IL-10 after 1 year of treatment. Furthermore, we explored the potential role of the baseline immunological profile as a biomarker to predict treatment response and found that, in the 40 patients with more severe disease (baseline CSMS > median), those with poor or no response to treatment had significantly higher baseline levels of IL-13 compared to highly responsive patients. Additionally, the analysis of effectiveness variables according to patients’ sensitization profiles showed similar outcomes, indicating that treatment with Acarovac Plus® was effective regardless of the predominant sensitization.

Immunological parameters can serve as surrogate measures of clinical response to AIT, playing a role as biomarkers to guide diagnosis and management, including treatment start, discontinuation, and reinstatement, and predict response to AIT.^17^ Given their value, guidelines recommend including changes in immunological parameters as outcomes to monitor AIT effects.^20^ Even though the immunological changes induced by AIT remain to be fully elucidated, investigators currently accept that AIT increases serum levels of allergen-specific IgG4, which blocks IgE-dependent, allergy-associated events, including histamine release and antigen presentation to T-cells, and transiently increases levels of allergen-specific IgE. Additionally, AIT is believed to switch the balance of T_H_1/T_H_2 responses towards a regulatory T_H_1 response, characterized by the release of cytokines IL-12, IL-10, and IFN-γ, among others.^15^, ^16^, ^17^ Given their relevance, this study focused on assessing these immunological parameters in patients receiving AIT with Acarovac Plus® to treat HDM allergy.

Regarding changes in IgE, the key mediators of allergic responses, the trend towards increased serum levels of DP-sIgE and significantly increased Der p 1-sIgE shown by the results of this study most likely reflected allergen exposure during AIT administration. Despite the heterogeneous results of previous studies, overall, it is accepted that DP-sIgE levels transiently increase during AIT treatment without clinical or functional relevance.^17^^,^^21^, ^22^, ^23^, ^24^, ^25^, ^26^, ^27^ While previous studies have mostly focused on DP-sIgE, our study further distinguished between the 2 major HDM allergens, Der p 1 and Der p 2, showing increased Der p 1-sIgE but not Der p 2-sIgE. In this regard, previous studies assessing conventional aluminum-formulated AIT reported transient increases in Der p 1-, Der p 2-, and Der p 23-sIgE after SCIT, suggesting higher allergenicity of this formulation compared to the MCT-associated allergoid used in this study.^7^^,^^28^ In addition, similar to previous studies assessing AIT with HDM allergens, our results showing a significant 2.2-fold increase in DP-sIgG4 one year after AIT confirm the accepted notion that AIT results in increased DP-sIgG4 and reflect the immunogenicity of the allergoid used in this study.^22^^,^^26^, ^27^, ^28^, ^29^ In this regard, the use of an allergoid instead of a native extract may have reduced the allergenicity while maintaining the immunogenicity of the allergen, resulting in modest increases of some, albeit not all, IgE and a concomitant increase in DP-sIgG4. Likewise, the use of MCT as an adjuvant, which, unlike other adjuvants (ie, aluminum), has been shown to curb the allergen-mediated induction of IgE, may explain the modest increase in IgE.^30^

Regarding cytokines as biomarkers of treatment effectiveness, the results from this study, which show a significant increase in IL-10 and a decrease, albeit not significant, in IL-5, are in line with the expected increase in T_H_1 responses at the expense of T_H_2 responses. Similarly, previous studies assessing AIT with HDM allergens have shown that allergen-induced IL-10 production paralleled a global suppression of T_H_2 proliferative responses and cytokine production.^31^ Importantly, IL-10 has been shown to suppress allergen-specific IgE secretion and increase sIgG4 production.^32^, ^33^, ^34^ Even though the relationship between changes in these parameters and clinical outcomes remains to be analyzed, the immunological changes observed in this study agree with the role of IL-10 and sIgG4 as mediators of immune tolerance, supporting the notion that immunotherapy restores a tolerant T-cell response.^31^

Due to the current indication of AIT in patients with moderate/severe symptoms and/or unresponsive to pharmacological treatment, we used the corresponding population of patients (ie, those with more severe symptoms) to evaluate immunological parameters as predictors of treatment response.^13^ Considering the exploratory nature of these analyses and to increase the ability of IL-13 to predict patients’ response, we excluded low responders (LRs) from the analysis and focused on the most extreme responders, non-responders (NRs) and high responders (HRs). We found that increased baseline levels of IL-13, IL-10, and T_H_2 cytokines (combined IL-4, IL-5, and IL-13) were associated with no response, albeit only IL-13 remained significantly different in the multivariate analysis, showing the potential value of IL-13 as predictor of treatment response. The production of IL-13 by allergen-specific T_H_2 CD4^+^ cells plays a role in allergen sensitization by switching antibody production to allergen-specific IgE. Conversely, during AIT-induced immune tolerance, the action of IL-13—together with that of other T_H_2 cytokines—is blunted by regulatory cytokines, including IL-10 and TGFβ.^16^^,^^35^ Considering the regulatory role of increased IL-10 in antagonizing T_H_2-mediated IgE responses during AIT-induced immune tolerance, increased baseline levels of IL-13 in NRs likely restrained the action of AIT-induced regulatory cytokines, limiting AIT clinical benefit.

Despite our comprehensive evaluation of immunological parameters as biomarkers of clinical response, serum immunoglobulins lacked a predictive value. Previous studies have shown that DP-sIgE, DP-sIgE/tIgE, and Der p 1-sIgE/Der p 1-sIgG4 may be useful biomarkers to predict clinical responses to AIT for HDM allergy, even though these results are yet to be confirmed.^22^^,^^25^^,^^27^^,^^36^^,^^37^ In this regard, our study and previous ones assessed immunoglobulin and cytokine levels using different samples (ie, serum and isolated peripheral blood mononuclear cells) and methods with different sensitivity thresholds. These methodological differences preclude direct comparisons among studies and may partially explain the heterogeneous results from the different studies, hampering the identification of validated biomarkers.

Marketed AIT products differ in contents, concentrations, and ratios of individual allergens, particularly those obtained from natural extracts, likely influencing the degree of response.^38^^,^^39^ In AIT for HDM, which contains different allergen molecules, the differences in the allergen composition of marketed AITs may further influence their ability to induce immune tolerance in patients with different sensitization profiles. Results obtained from this study, which show no differences between changes in effectiveness variables according to the predominant sensitization, indicate that Acarovac Plus® is effective regardless of the sensitization profiles represented in this study. Similar to this, previous studies assessing other AITs also reported similar effectiveness in patients with different sensitization profiles.^7^^,^^40^

Despite its prospective nature, the results from this study should be interpreted in the context of some limitations related to its real-life setting. The inclusion of all patients starting AIT regardless of their immunological profile resulted in an uneven distribution of patient groups for analysis, with a reduced number of patients in some categories (ie, predominant sensitization groups). Additionally, the detection of serum levels of cytokines, which are typically low, was constrained by the methods used in real life, precluding the use of more sensitive methods frequently used in clinical trials and potentially limiting the sensitivity of our analyses. Likewise, Der p 23-IgE, Der p 1-sIgG4 and Der p 2-sIgG4 are not routinely assessed in the Spanish clinical practice and few centers have their laboratories equipped to determine serum concentrations of these immunoglobulins. For this reason, IgG4 specific to the DP allergens and sensitization to Der p 23, despite being a major allergen, were not assessed in this study. Furthermore, while SCIT with HDM allergen extracts is typically administered for 3 years, patients were assessed after one year of AIT, precluding the analysis of immunological parameters after prolonged periods of treatment. In spite of these limitations, the size of the study was large and was able to capture immunological response in a population mirroring the sensitization profiles of patients from different centers, encompassing different geographical areas. Unlike clinical trials, this study lacked strict selection criteria, allowing to capture the effects of Acarovac Plus® in the heterogeneous population found in routine clinical practice. This study confirms that Acarovac Plus® partly summarizes previously described immunological responses to other AIT treatments and provides useful information for clinicians to stratify patients and optimize AIT prescription and clinical management of patients with allergy to HDM. Furthermore, our finding that IL-13 may be a potentially valuable biomarker to predict treatment response in non-responsive and hyper-responsive patients warrants future larger studies.

## Conclusion

Our results obtained from a cohort of patients with allergic rhinitis with and without asthma treated in the real-world setting provide evidence of increased levels of DP-sIgG4, Der p 1 sIgE, and IL-10 as mechanisms of immune tolerance induced by AIT specific for HDMs. In addition, increased baseline levels of IL-13 in non-responders suggest that this immunological parameter might serve as biomarker to predict clinical response. Finally, our study suggests that patients sensitized to different DP allergens may benefit from treatment with an MCT-associated house dust mite allergoid.

## Abbreviations

AIT, allergen immunotherapy; ANOVA, analysis of variance; AR, allergic rhinitis; CI, confidence interval; CSMS, combined symptom and medication score (CSMS); Der p 1-sIgE, Der p 1-specific IgE; Der p 2-sIgE, Der p 2-specific IgE; DP, Dermatophagoides pteronyssinus; DP-sIgE, Dermatophagoides pteronyssinus-specific IgE; DP-sIgG4, Dermatophagoides pteronyssinus-specific IgG4; HDM, house dust mite; HRs, high responders; IQR, interquartile range (Q1, Q3); LRs, low responders; MCT, MicroCrystalline Tyrosine; NIPF, nasal inspiratory peak flow; NPT, nasal provocation test; NRs, non-responders; OR, odds ratio; SC/NP, same class/no predominant sensitization; SD, standard deviation; VAS, visual analog scale.

## Agreement to publish the work

All authors have reviewed and approved the final version of the manuscript.

## Author contributions

JLJ, AB and CT-B made substantial contributions to the conception and design of the work; and the analysis, and interpretation of data for the work. CP, AR, FM, MJR, AP, AV, AM and AT made substantial contributions to the acquisition of data for the work. JLJ and CT-B drafted the article and CP, AR, FM, MJR, AP, AV, AM, AT and AB contributed to revising the work critically for important intellectual content. All authors gave their final approval of the version to be published, and agree in their accountability for all aspects of the work in ensuring that questions related to the accuracy or integrity of any part of the work are appropriately investigated and resolved.

## Ethics approval and consent to participate

All included patients and legal representatives of patients <18 years signed a written informed consent before any data was recorded. The study was conducted in accordance with the Helsinki Declaration and the local Personal Data Protection Law (LOPD 15/1999); the study protocol was approved by the Ethics Committee of Hospital Germans Trias i Pujol (Barcelona, Spain) (EPA-14-023).

## Editorial policy confirmation and agreement

I hereby state that the manuscript, including related data, figures and tables, has not been previously published and has not been submitted to any other journal (in any other language or any other type of publication), either by me or any of my co-authors.

## Availability of data and materials

The anonymized datasets collected during the current study are available from the corresponding author on reasonable request.

## Declaration of competing interest

This work was supported by Allergy Therapeutics Ibérica (Sant Joan Despí, Barcelona, Spain). JLJ, AB and CT-B are Allergy Therapeutics employees; CP has collaborated with Allergy Therapeutics Ibérica to attend courses/congresses/medical training; AR reports personal fees from Allergy Therapeutics, during the conduct of the study; personal fees from Leti, Stallergenes, Roxall, Diater, and Hal, outside the submitted work; MJR reports personal fees from Astra-Zeneca, GSK, Allergy Therapeutics, and Chiesi, outside the submitted work; FM, AP, AV, AM and AT have nothing to disclose.
