# Supplementary material for: Immunological parameters as biomarkers of response to MicroCrystalline Tyrosine-adjuvanted mite immunotherapy
Source: World Allergy Organ J. 2021 Jun 6;14(6):100545. doi: 10.1016/j.waojou.2021.100545 (PMC8192729; doi:10.1016/j.waojou.2021.100545)
Supplement: Multimedia component 1 [file mmc1.doc]

| **Table S1.** Baseline effectiveness variables according to predominant sensitization, *mean (SD)* | | | | | | |
| --- | --- | --- | --- | --- | --- | --- |
|  | **Baseline Values** | | | ***P* Value^†^** | | |
|  | **Der p 1 n=11^‡^** | **Der p 2 n=37** | **NP n=51** | **Der p1 vs*.* Der p2** | **Der p 1 vs. SC/NP** | **Der p 2 vs. SC/NP** |
| CSMS | 1.74 (1.17) *n=11* | 1.64 (1.1) *n=36* | 1.49 (0.96) *n=43* | 0.754 | 0.644 | 0.768 |
| Daily symptom score | 0.95 (0.74) *n=11* | 0.76 (0.67) *n=36* | 0.93 (0.65) *n=43* | 0.482 | 0.898 | 0.147 |
| Daily medication score | 0.78 (0.64) *n=11* | 0.84 (0.74) *n=36* | 0.54 (0.61) *n=46* | 0.870 | 0.188 | **0.034** |
| Well days (%) | 36.0 (34.1) *n=11* | 43.5 (34.5) *n=36* | 50.3 (39.9) *n=46* | 0.537^§^ | | |
| Bad days (%) | 17.5 (27.4) *n=11* | 16.2 (27.0) *n=36* | 15.6 (23.1) *n=45* | 0.703^§^ | | |
| NPT (% drop of NIPF) | 50.0 (18.7) n=10 | 38.4 (24.0) n=35 | 33.1 (22.5) n=51 | 0.189 | **0.034** | 0.319 |
| NPT (symptom score) | 5.91 (2.88) *n=11* | 5.62 (2.1) *n=37* | 6.25 (1.82) *n=51* | 0.618 | 0.800 | 0.155 |
| ESPRINT-15 | 3.0 (1.1) *n=11* | 2.56 (1.61) *n=36* | 2.67 (1.71) *n=49* | 0.377 | 0.443 | 0.811 |
| VAS | 7.39 (2.01) n=11 | 7.33 (2.32) n=37 | 6.07 (2.36) n=50 | 0.941 | 0.097 | **0.005** |
| CSMS, combined symptom and medication score; NIPF, nasal inspiration peak flow; NP, non-predominant sensitization; NPT, nasal provocation test; SC/NP, same class/no predominant sensitization; SD, standard deviation; VAS, visual analog scale  **^†^**Mann-Whitney test  **^‡^**Total number of patients per group; the number of patients with available data at each timepoint and treatment are indicated in italics in the corresponding cell  ^§^Fisher’s test, comparing the three groups | | | | | | |

# Supplemental Table

#
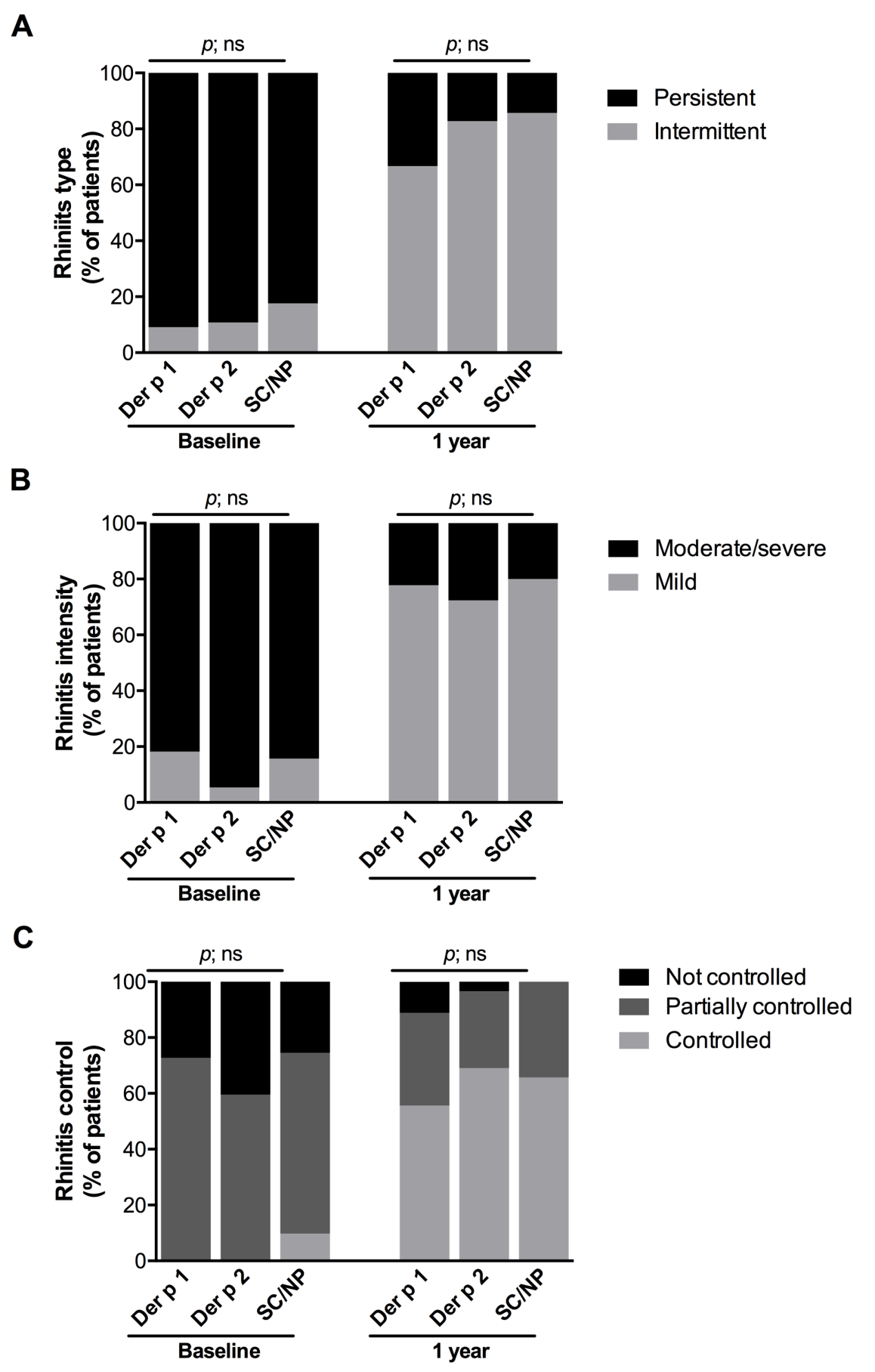
Supplemental Figure

**Figure S1**. Rhinitis frequency (A) and severity (B) according to the ARIA classification and degree of rhinitis control (C) at baseline and one year after treatment with AIT. Columns represent the distribution of patients (%) in the different categories for each of the predominant sensitization groups. SC/NP, same class/no predominant sensitization. McNemar test, *p*; ns (non-significant).
